# Tick-borne encephalitis related uveitis: a case report

**DOI:** 10.1186/s12886-021-02068-1

**Published:** 2021-08-28

**Authors:** Nafsika Voulgari, Claire-May Blanc, Vanessa Guido, Daniele C. Rossi, Yan Guex-Crosier, Florence Hoogewoud

**Affiliations:** 1 Department of Ophthalmology, University of Lausanne, Jules-Gonin Eye Hospital, Fondation Asile des Aveugles, avenue de France 15, 1002 Lausanne, Switzerland; 2 Department of Internal Medicine, Etablissements Hospitaliers du Nord Vaudois, 1400 Yverdon-les-Bains, Switzerland; 3 Department of Neurology, Centre Hospitalier Universitaire Vaudois, 1011 Lausanne, Switzerland

**Keywords:** Infectious disease, Infectious uveitis, Tick-born encephalitis, Uveitis, Viral uveitis, Virus, Case report

## Abstract

**Background:**

Tick-borne encephalitis (TBE) is an infectious disease of the central nervous system caused by the TBE virus (TBEV), which is usually transmitted by a tick-bite, with increasing incidence in northeastern Europe and eastern Asia during the past decade. Ocular involvement has not been described in the literature to date.

**Case presentation:**

A 58-year-old patient presented to the emergency department with occipital headaches and poor balance for 5 days. He reported a tick-bite 6 weeks before without erythema migrans followed by a flu-like syndrome. Serological testing was negative for Borreliosis and TBEV. At presentation, he was febrile with neck stiffness and signs of ataxia. Three days later, he presented unilateral visual loss in his right eye. Examination revealed non granulomatous anterior uveitis, vitreous inflammation, and retinal haemorrhages at the posterior pole without macular oedema or papillitis. Polymerase chain reaction (PCR) of the cerebrospinal fluid returned negative for all Herpes family viruses. No clinical evidence of other infection nor malignancy was identified. A seroconversion of the TBEV- immunoglobulin titres was observed 2 weeks later while the serum antibodies for Borrelia were still not detected. Magnetic resonance imaging was unremarkable. We concluded to the diagnosis of TBE-related uveitis. Under supportive treatment, there was complete resolution of the neurological symptoms and the intraocular inflammation without sequelae within the following weeks.

**Conclusions:**

We describe a new association of TBEV with uveitis. In view of the growing number of TBE cases and the potential severity of the disease we aim at heightening awareness to achieve prompt recognition, prevention, and treatment.

## Background

Tick-borne encephalitis (TBE) is a severe infection of the central nervous system caused by the TBE virus (TBEV), a member of the flavivirus genus, endemic in northeastern Europe and eastern Asia [1]. TBEV can be transmitted by a tick-bite with the highest incidence in the summer months, although transmission through consumption of unpasteurized milk products has also been documented. In Switzerland, as in other European countries, a significant rise in incidence has been observed over the past decade (1.2 versus 5.6 cases per 100′000 persons in 2010 versus 2020) [2, 3]. In view of this upsurge of cases, TBE infections are regarded as a growing public health problem.

Clinical presentation of TBE typically consists of a biphasic pattern. After an incubation period of approximately 8 days, a flu-like syndrome ensues for a few days, followed by an asymptomatic stage of 1 week. One third of patients progress to a second phase of neurological manifestations, including meningitis, encephalitis or myelitis [1]. Several clinical and laboratory factors have been linked to a more severe course of the disease and poor prognosis [4]. The overall case fatality ratio is estimated at 0.5% but can reach 40% in case of infection by the TBEV far eastern subtype [2]. To our knowledge, ocular involvement has not been described in the literature to date. We herein present a case of uveitis associated with TBE.

## Case presentation

A 58-year-old man with an unremarkable medical history presented to the emergency department with occipital headaches associated with photophobia, phonophobia, nausea, fatigue, and poor balance for 5 days. The patient reported a tick bite 6 weeks before without erythema migrans. A flu-like syndrome followed ten days after and rapidly resolved without treatment. At this time, he had undergone serological testing by his general practitioner which was negative for Borreliosis and TBEV. At presentation, he was febrile with neck stiffness and signs of ataxia and was admitted for further investigation.

**Fig. 1:**
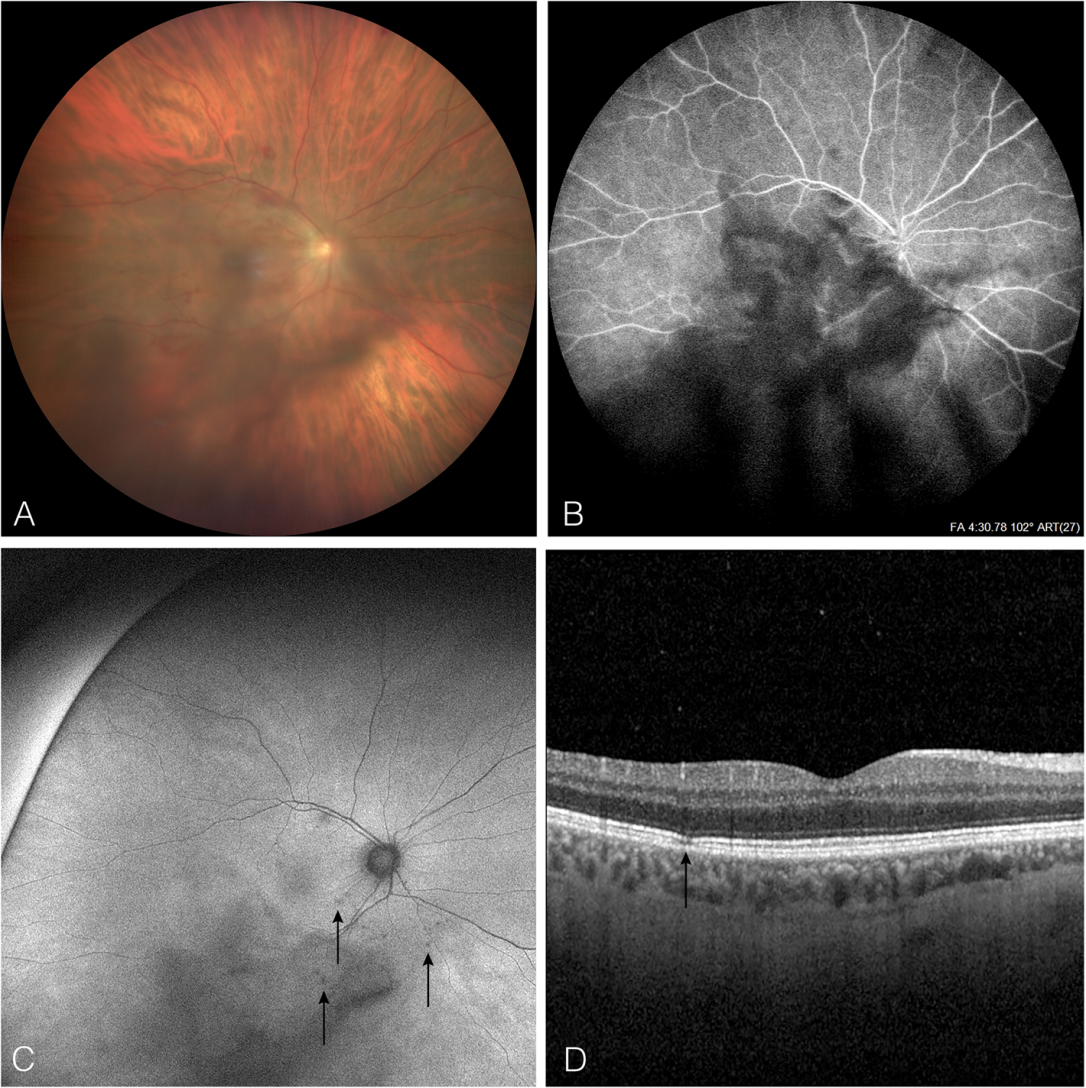
**A** Fundus photograph of the right eye demonstrating vitreous inflammation and retinal haemorrhages. **B** Fundus fluorescein angiography (FFA) showing the absence of macular oedema and papillitis and the presence of vitritis. **C** Fundus (FAF) 5 days after the FFA revealing small hypoautofluorescent lesions around the optic nerve (arrows) and outer retinal lesions on optical coherence tomography (OCT) (arrow). **D** FAF and OCT images were not previously exploitable, at the time of FFA, as the presence of vitritis impeded fundus visualisation

Three days later, he presented unilateral visual loss in his right eye (RE). Visual acuity was 20/100 in the RE and 20/20 in the left eye (LE). Intraocular pressure was normal. Slit lamp examination of the RE revealed non granulomatous anterior uveitis with 2+ cells. Fundus examination showed 2+ of vitreous haze and cells, flame-shaped and dot retinal haemorrhages at the posterior pole (Fig. 1A) (CLARUS™ 500, Carl Zeiss Meditec AG, Germany). The LE was unremarkable. Fundus fluorescein angiography (FFA), indocyanine green angiography (ICG) and optical coherence tomography (OCT) (Spectralis, Heidelberg Engineering, Heidelberg, Germany) excluded the presence of macular oedema or papillitis (Fig. 1B). When the vitritis started to improve and a better analysis of the retina was possible, discrete hypoautofluorescent lesions were observed and appeared as outer retinal lesions on OCT (Fig. 1C and D). We concluded a diagnosis of unilateral panuveitis. Topical treatment by prednisolone acetate 1% six times per day was introduced for the treatment of the anterior uveitis. To cover for a possible herpes meningoencephalitis with retinal involvement, an antiviral therapy with intravenous acyclovir 10 mg/kg/TID was initiated.

Cerebrospinal fluid (CSF) examination revealed elevated leukocyte counts (81 cells/mm3) with mononuclear cell dominance and an elevated protein level (1030 μmol/l). Polymerase chain reaction (PCR) returned negative for all Herpes family viruses and acyclovir was therefore discontinued. Human Immunodeficiency Virus (HIV) and syphilis were equally negative, without any other clinical evidence of such infection being identified and no malignant cells were demonstrated. Serum antibodies for Borrelia were still not detected whereas a seroconversion of the TBEV- immunoglobulin (Ig) titres measured by Enzyme Immunoassay was noted 2 weeks after admission (Index of IgM at 2.0 and IgG at 4.9 for a norm of 0.8–1.2 and 07.1.3 respectively). Magnetic resonance imaging (MRI) was unremarkable. Meanwhile the patient’s neurological condition gradually deteriorated with the onset of psychomotor retardation, aphasia and hallucinations. The patient remained hospitalised and supportive care was pursued.

The neurological symptoms fully resolved within 3 weeks. A complete resolution of the intraocular inflammation without sequelae was observed at 5 weeks with a visual acuity back to normal (20/16).

## Discussion and conclusions

To our knowledge, this is the first report of a TBEV related uveitis. The diagnosis was based on the typical neurological manifestations, serological tests, the exclusion of any alternative diagnosis and the rapid spontaneous resolution of the inflammation without treatment.

TBEV belongs to the flavivirus genus, a family of viruses that includes amongst others West Nile Virus, Dengue virus, Japanese Encephalitis virus, Yellow fever virus and Zika virus [5]. Flaviviruses have been related to a spectrum of inflammatory ocular manifestations, ranging from anterior uveitis in Zika virus [6] to multifocal chorioretinitis in West Nile virus [7]. To our knowledge, this is the first report of TBE-associated ocular involvement. However, a case of intermediate uveitis after TBE vaccine has been described [8].

The steep increase in incidence of TBE infections in Europe and Asia over the past two decades is related to an upsurge of cases, not only in established foci but also the appearance of new foci. A seasonal distribution is seen with the highest prevalence being reported from spring until November [1]. This is associated with the period of highest tick activity, together with increased human outdoor activity during the warmer months. The rise in the reported incidence is considered to be multifactorial. Environmental factors include climate change with global warming and raised abundance of the tick vector, as well as socioeconomic aspects comprising the growth of tourism. Heightened medical awareness, greater surveillance and effective diagnostics will also play an important role [1].

In conclusion, this report aims at describing a new association of TBEV with uveitis and at increasing the awareness of this infection in view of the increase of cases and the potential severity of the disease.

## Data Availability

All data generated and analyzed during this study are included in this article.

## Abbreviations

TBE: Tick-borne encephalitis
TBEV: Tick-borne encephalitis virus
RE: Right eye
LE: Left eye
FFA: Fundus fluorescein angiography
ICG: Indocyanine green angiography
OCT: Optical coherence tomography
CSF: Cerebrospinal fluid
PCR: Polymerase chain reaction
HIV: Human Immunodeficiency Virus
Ig: Immunoglobulin
MRI: Magnetic resonance imaging

## References

[1] Lindquist L, Vapalahti O (2008). Tick-borne encephalitis.

[2] Beauté J, Spiteri G, Warns-Petit E, Zeller H (2018). Tick-borne encephalitis in europe, 2012 to 2016. Eurosurveillance..

[3] Schuler M, Zimmermann H, Altpeter E, Heininger U (2014). Epidemiology of tick-borne encephalitis in Switzerland, 2005 to 2011. Eurosurveillance..

[4] Radzišauskienė D, Urbonienė J, Kaubrys G, Andruškevičius S, Jatužis D, Matulytė E, et al. The epidemiology, clinical presentation, and predictors of severe Tick-borne encephalitis in Lithuania, a highly endemic country: A retrospective study of 1040 patients. PLoS One. 2020;15. 10.1371/journal.pone.0241587. 10.1371/journal.pone.0241587 PMC7676731 33211708

[5] Singh S, Kumar A (2018). Ocular manifestations of emerging flaviviruses and the blood-retinal barrier. Viruses..

[6] Furtado JM, Espósito DL, Klein TM, Teixeira-Pinto T, da Fonseca BA (2016). Uveitis associated with Zika virus infection. N Engl J Med.

[7] Khairallah M, Ben Yahia S, Ladjimi A, Zeghidi H, Ben Romdhane F, Besbes L (2004). Chorioretinal involvement in patients with West Nile virus infection. Ophthalmology..

[8] Ness T, Boehringer D, Heinzelmann S (2017). Intermediate uveitis: pattern of etiology, complications, treatment and outcome in a tertiary academic center. Orphanet J Rare Dis.

